# Magnetic nanocarriers as a therapeutic drug delivery strategy for promoting pain-related motor functions in a rat model of cartilage transplantation

**DOI:** 10.1007/s10856-021-06508-8

**Published:** 2021-03-31

**Authors:** Xingyu Zhang, Jianjun Yang, Baochang Cheng, Shenli Zhao, Yao Li, Hui Kang, Shiyi Chen

**Affiliations:** 1 Department of Sports Medicine, Huashan Hospital, Fudan University, Shanghai, 200040 China; 2 Department of Orthopaedics, Tenth People’s Hospital, Tongji University, Shanghai, 200072 China; 3 State Key Laboratory of Molecular Engineering of Polymers, Fudan University, Shanghai, 200433 China; 4 Department of Macromolecular Science, Fudan University, Shanghai, 200433 China; 5 Department of Joint Surgery, Yangpu Hospital Affiliated to Tongji University, Shanghai, 200082 China; 6 Department of Orthopaedics, Tenth People’s Hospital, Nanjing Medical University, Shanghai, 200072 China

## Abstract

Cartilage is an avascular tissue with low cellularity and insufficient self-repair response. In clinical practice, a large articular cartilage defect is usually fixed by cartilage transplantation. Importantly, the fast repair process has been demanded postoperatively in the area between the host cartilage and the transplanted cartilage. In the past few years, magnetic nanoparticles have drawn great attention due to their biocompatible, biodegradable, and nontoxic properties. In addition, the nanoparticles can easily pass through the cell plasma membrane and increase the cellular uptake efficiency. Here, a therapeutic drug delivery strategy was proposed for cartilage repair. The prepared kartogenin (KGN)-conjugated magnetic nanocarriers (KGN@NCs) promoted the viability of chondrocytes in vitro. In a rat model of cartilage transplantation, intra-articularly delivered KGN@NCs generated cartilage with a flat surface and a high level of aggrecan in vivo. Notably, KGN@NCs were also capable of improving the pain-related motor functions. They promoted the motor functional parameters including the print area and intensity to restore to a normal level compared with the single KGN. Therefore, these therapeutic drug nanocarriers provided the potential for cartilage repair.

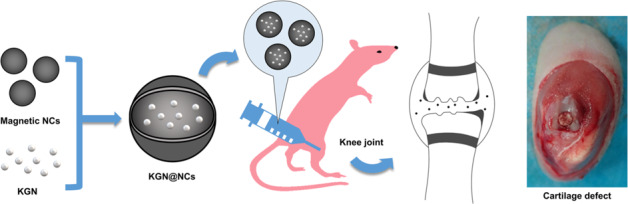

## Introduction

Cartilage, a kind of avascular tissue only with one cell type of chondrocytes, lacks self-repair response [1, 2]. Trauma, overuse, and aging are the main causes for articular cartilage damage that results in joint swelling, pain, and subsequent osteoarthritis, which is affecting hundreds of millions of people around the world [3–6]. From a surgical perspective, about 60% of the patients who receive arthroscopic examinations have articular cartilage lesions, of which 67% are identified as focal defects [7]. There have been estimated 2,000,000 patients around the USA who underwent surgical procedures for articular cartilage defects annually in the past few years [8]. The traditional surgical treatments for these cartilage defects include bone marrow stimulation, autograft transplantation, and allograft transplantation [9, 10]. However, the therapeutic effect of bone marrow stimulation is minor for a large defect that leads to fibro-cartilage formation. Allograft needs the additional storage that results in cell viability loss and disease transmission. Thus, surgeons mostly choose autograft transplantation to fix large cartilage defects. This procedure has potential advantages, as it involves easier process and lower risk. Moreover, autograft transplantation helps to maintain original biological hyaline-like cartilage [11]. Nevertheless, minimal autografts are more desirable to avoid the complications of the donor area when the transplantation is performed [12]. Meanwhile, fast postoperative repair of the area between the host cartilage and the transplanted cartilage is urgently demanded after the surgery.

Therapeutic nanoparticles have been emerging in the biomedical material designing [13]. As for the clinical applications, these nanoparticles are preferable for many necessary properties such as good compatibility, long circulating time, high accumulation in the target tissues, and efficient drug loading and releasing. However, ideal therapeutic strategies of the nanoparticles for clinical drug delivery are still required. In recent years, magnetic iron oxide nanoparticles have drawn great attention owing to the excellent biocompatible, biodegradable, and nontoxic properties [14–16]. They are easily controlled by external magnetic attraction for isolation and manipulation. Notably, these magnetic nanoparticles can readily pass through the plasma membrane of the natural cells to increase the cellular uptake efficiency. Besides, the iron in the magnetic nanoparticles can also act as a nutrient to be metabolized, thereby suitable for in vivo applications [17]. Most importantly, unlike other organic or inorganic nanoparticles, magnetic nanoparticles have been applied in clinical practice for more than 10 years. They are safe and appropriate for biological imaging as well as various pathophysiological conditions [18–21]. These above advantages are benefit for magnetic nanoparticles to work as therapeutic drug nanocarriers (NCs).

Recently, a small molecular drug kartogenin (KGN) was found effective for cartilage repair [22]. In brief, this drug has the properties to maintain biological characteristics of cartilage and promote chondrocyte regeneration in vitro. In addition, it was demonstrated to be chondroprotective in osteoarthritis animal models. Up to now, many studies have followed this research and attempted to promote cartilage repair with various approaches [23–27]. For example, KGN-grafted hydrogel scaffold, collagen nanofibers encapsulated with KGN, and stem cell-related therapy were the representative therapeutic strategies involving magnetic nanoparticles in the past few years [28–32]. Hence, KGN is a promising candidate for the fast repair of the damaged cartilage. However, a regional cartilage defect is not equivalent to the broad lesion in osteoarthritis, the efficient transportation of KGN into the target cells of the area between the host cartilage and the transplanted cartilage is the key to promote postoperative cartilage repair.

**Fig. 1:**
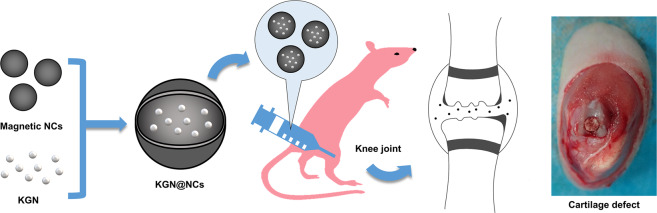
The graphical abstract of the preparation and application of KGN@NCs for cartilage repair. The synthesized KGN@NCs were intra-articularly injected into the knee joint in a rat model of cartilage transplantation and subsequently promoted the cartilage repair process

In this study, a therapeutic drug delivery strategy was proposed for cartilage repair, which consisted of KGN-conjugated magnetic NCs (KGN@NCs). As shown in Fig. 1, the synthesized KGN@NCs not only easily loaded and released KGN but also efficiently facilitated the cellular uptake. Then, in a rat model of cartilage transplantation, we hypothesized that the intra-articularly delivered KGN@NCs would enhance the repair process of the area between the host cartilage and the transplanted cartilage and improve the motor functions of the rats. This strategy provided knowledge for the development of the relevant therapeutic NCs in future.

## Methods

### Materials and animals

Iron chloride hexahydrate, trisodium citrate dihydrate, ethylene glycol, and ethanol were purchased from Sinopharm Chemical Reagent Co. Ltd (Shanghai, China). KGN was purchased from Sigma-Aldrich Inc (St. Louis, USA). Toluidine blue staining kit was purchased from Solarbio Life Sciences (Beijing, China). Cell counting kit-8 (CCK-8) was purchased from Dojindo Molecular Technologies Inc (Kumamoto, Japan). Cy5 amine dye was purchased from ApexBio Technology (Houston, USA). DAPI dye and enzyme-linked immunosorbent assay (ELISA) kits were purchased from Beyotime Biotechnology (Shanghai, China). All the rats were provided by Department of Laboratory Animal Science of Fudan University, and the use of the animals was approved by Animal Care and Use Committee of Fudan University. During the experiment, all the rats were fed a standard rat chow and drank water ad libitum.

### Preparation and characterization of KGN@NCs

The iron oxide NCs were prepared with a modified solvothermal reaction. Briefly, 4 mmol iron chloride hexahydrate was dissolved with 0.8 mmol trisodium citrate dihydrate in ethylene glycol. The mixture was heated at 200 °C for 12 h in a Teflon-lined stainless-steel autoclave. The resulting product (NCs) was alternately washed with ethanol and water for several times after cooling down and separated with a magnet. KGN was encapsulated into NCs in a tube rotator (DLAB Scientific, China) for 24 h to fabricate KGN@NCs. Then, a transmission electron microscope (TEM, JEOL, Japan) was used for the morphology characterization of NCs, and a Zetasizer analyzer (Malvern Panalytical, U.K.) was used for dynamic light scattering (DLS) measurement of NCs and KGN@NCs.

### Drug loading and releasing test

NCs and KGN@NCs were, respectively, resuspended with phosphate buffer saline (PBS) in a dialysis bag (cut-off 14,000). A UV–vis spectrophotometer (PerkinElmer, USA) was used to acquire the UV–vis absorption spectra of NCs and KGN@NCs. The loading efficiency was as follows: entrapped KGN (wt)/KGN@NCs (wt) × 100%. Then, KGN@NCs were incubated in a dialysis bag in 200 mL PBS at pH 7.4 and pH 6.5 (37 °C, 120 rpm). At 0, 15, and 30 min, 1, 2, 4, 6, 8, 12, and 24 h after incubation, the UV–vis absorbance was respectively measured. Besides, the correlation between the KGN level and UV–vis absorbance was also calculated.

### In vitro cytocompatibility and proinflammatory test

Chondrocytes were isolated from the articular cartilage of a skeletally mature rat and characterized by toluidine blue staining. PBS, KGN, and KGN@NCs were, respectively, cocultured with chondrocytes (1 × 10^5^ cells/mL) in cell culture plates. The absorbance at 450 nm of CCK-8 after incubation was recorded with a multimode reader (BioTek, USA). Then, KGN@NCs were labeled with Cy5 and incubated with chondrocytes for 2 h, and the cell nuclei were labeled with DAPI dye. The fluorescent image was captured with a laser scanning confocal microscope (LSCM, Leica, Germany). Afterward, PBS and KGN@NCs were incubated with macrophages (1 × 10^5^ cells/mL) isolated from the abdominal cavity of a rat for 2 h. The levels of tumor necrosis factor-α (TNF-α) and interleukin-1β (IL-1β) in the culture medium were determined with ELISA kits.

### The animal model of cartilage transplantation

Aseptically, the rats were intraperitoneally anesthetized with pentobarbital sodium (5 mg/100 g). The left femoral trochlea of the joint was exposed. An orthotopic cartilage transplantation with a size of 2.5 mm was made on the left. Then, the bleeding was stopped and the wound was sutured layer by layer. Afterward, all the rats were randomly assigned into three groups (*n* = 6), which were PBS group, KGN group, and KGN@NCs group. They were intra-articularly injected with PBS, KGN, and KGN@NCs for three times in the first week with 10 μM KGN in 50 μL volume, respectively.

### Gait analysis

The gait parameters were acquired at 4 weeks after the surgery to evaluate motor functions with Catwalk gait analyzing system (Noldus, The Netherlands). The rats were trained to pass through the walkway before collecting the gait parameters. Several parameters including print area, max contact area, max contact mean intensity, and max contact max intensity were analyzed.

### Histological assessment

The rats were sacrificed at 4 weeks after the surgery. The knee joints were carefully taken out and fixed with 10% formaldehyde for 48 h. After the decalcification process at 37 °C for 20 days, the joints were sliced into 5-μm-thick sections for hematoxylin and eosin (HE) staining and safranin O/fast green staining. Besides, O’Driscoll scoring system for cartilage repair was used in this study to evaluate the repaired cartilage [33].

### Statistical analysis

All data were presented as mean ± standard deviation (SD). GraphPad Prism 8 (GraphPad Software, USA) was used for the statistical analysis with one-way or two-way ANOVA and Tukey’s multiple comparisons. Statistically significant was considered when a *p* < 0.05, and ns meant no statistical significance.

## Results and discussion

### Preparation and characterization of KGN@NCs

**Fig. 2:**
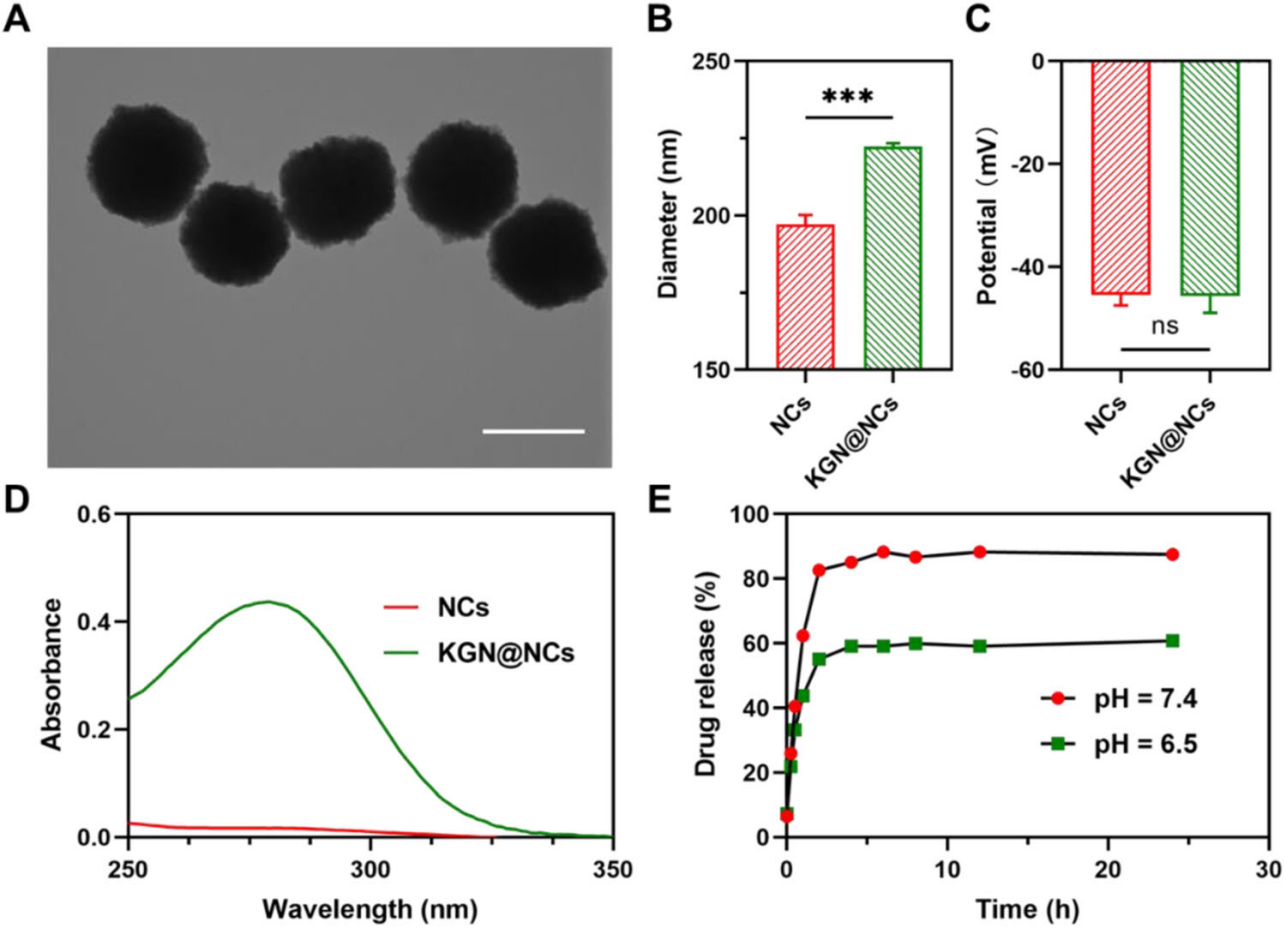
The preparation and characterization of KGN@NCs. **A** TEM image of the prepared NCs (scale bar, 200 nm). DLS measurement for the sizes (**B**) and zeta potentials (**C**) of NCs and KGN@NCs (*n* = 3). Data were presented as mean ± SD. ***p < 0.001. **D** UV–vis absorption spectra for KGN of the dialysis solution of NCs and KGN@NCs in PBS. **E** Representative levels of the accumulative KGN release of KGN@NCs at pH 7.4 and pH 6.5

The morphology of the prepared NCs was characterized with TEM, which showed a uniform structure of ~200 nm (Fig. 2A). DLS measurement in PBS also revealed an average size of 197.20 ± 2.94 nm (Fig. 2B) of NCs with a zeta potential of −45.47 ± 2.00 mV (Fig. 2C). Then, KGN was encapsulated into NCs to fabricate KGN@NCs by blending them together in a rotator. The resulting KGN@NCs was of 222.30 ± 1.09 nm size on average that increased by 25.10 nm (*p* < 0.001), but the zeta potential of −45.70 ± 3.23 mV showed no significant difference compared with NCs. Furthermore, the successful encapsulation of KGN was confirmed by an obvious UV–vis absorption peak of KGN at ~280 nm of KGN@NCs than that of NCs (Fig. 2D). These results indicated that KGN@NCs was successfully synthesized with a uniform structure.

To investigate the capability of KGN@NCs to release KGN, the linear regression of KGN concentration (*x)* and UV–vis absorbance (*y*) was calculated to be y = 81.91 × *x* (Fig. S1). It was reported that the damaged cartilage experiences an acid–base range from pH 6.5 to pH 7.4 in some pathological conditions, among which the acidic condition is more difficult for cartilage repair [34]. Thus, drug release test was further conducted in acidic and basal conditions. About 87.52% KGN was released at pH 7.4 and about 60.78% was released at pH 6.5 (Fig. 2E). It is considered that the difference of the accumulative drug release rates between these pH conditions was attributed to the existence of a carboxylic group in KGN molecule [23], which might be inhibited to disassemble during the process of dissociation at pH 6.5 rather than pH 7.4. Therefore, the synthesized KGN@NCs were capable of loading and releasing KGN in complex environments.

### Cytocompatibility and proinflammatory tests

**Fig. 3:**
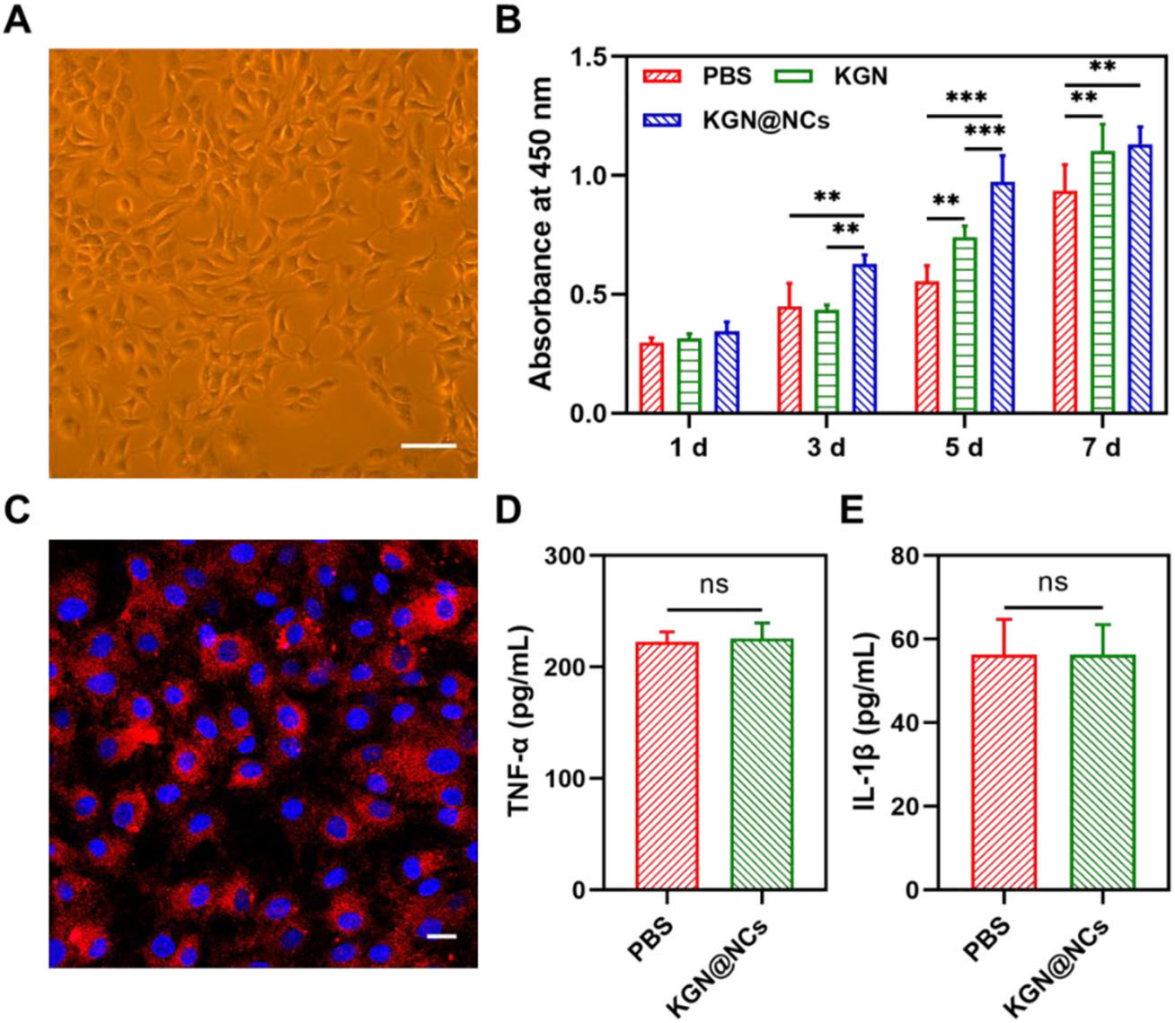
The cytocompatibility and proinflammatory activity of KGN@NCs. **A** Chondrocytes isolated from the articular cartilage (scale bar, 100 μm). **B** CCK-8 assay of the chondrocytes cocultured with PBS, KGN solution, and KGN@NCs, respectively (*n* = 6). **C** Representative LSCM for the evaluation of the cellular uptake of Cy5-labeled KGN@NCs in chondrocytes after incubation for 2 h (scale bar, 20 μm). ELISA for TNF-α (**D**) and IL-1β (**E**) concentrations of macrophages that were incubated with PBS and KGN@NCs for 2 h (*n* = 6). All data were presented as mean ± SD. **p < 0.01, ***p < 0.001

Chondrocytes were isolated from the articular cartilage of a skeletally mature rat (Fig. 3A), which were characterized by toluidine blue staining (Fig. S2). Then, the viability of chondrocytes was tested by CCK-8 assay after coculturing with PBS, KGN solution, and KGN@NCs, respectively. The absorbance at 450 nm of KGN@NCs group was significantly higher than that of PBS group at 3 days (*p* < 0.01), 5 days (*p* < 0.001), and 7 days (*p* < 0.001) (Fig. 3B), which indicated that the viability of chondrocytes was promoted after treating with KGN@NCs, thus there was minimal cytotoxicity of KGN@NCs. Furthermore, KGN@NCs group also showed an increased absorbance of 1.45-fold at 3 days (*p* < 0.01) and 1.31-fold at 5 days (*p* < 0.001) than KGN group. Thus, KGN@NCs promoted the viability of chondrocytes more efficiently compared with the single KGN.

Furthermore, the cellular uptake of KGN@NCs was assessed by visualization of chondrocytes after incubation for 2 h with LSCM. Most of chondrocytes showed obvious red fluorescence from Cy5 around blue fluorescence from DAPI (Fig. 3C). The majority of the red fluorescence was not overlapped with the blue fluorescence, which implied that KGN@NCs mainly distributed in the cell plasma. Importantly, the bioconjugation site of KGN with the target cells was previously reported to locate in the cell plasma as well [22]. Therefore, the prepared KGN@NCs delivered KGN into its target sites to promote the viability of the cells.

Next, the reaction of immune system to KGN@NCs was required to be studied before further applications. Herein, macrophages were used to check the proinflammatory activity of KGN@NCs, and several inflammatory factors were examined by ELISA. There was no significant difference in the concentrations of both TNF-α and IL-1β between PBS group and KGN@NCs group (*p* > 0.05) (Fig. 3D, E). The above results showed that these biocompatible KGN@NCs had the biosafe property and no obvious proinflammatory activity, which were beneficial to the subsequent in vivo studies of the effects for cartilage repair.

### Gait analysis for motor functions

**Fig. 4:**
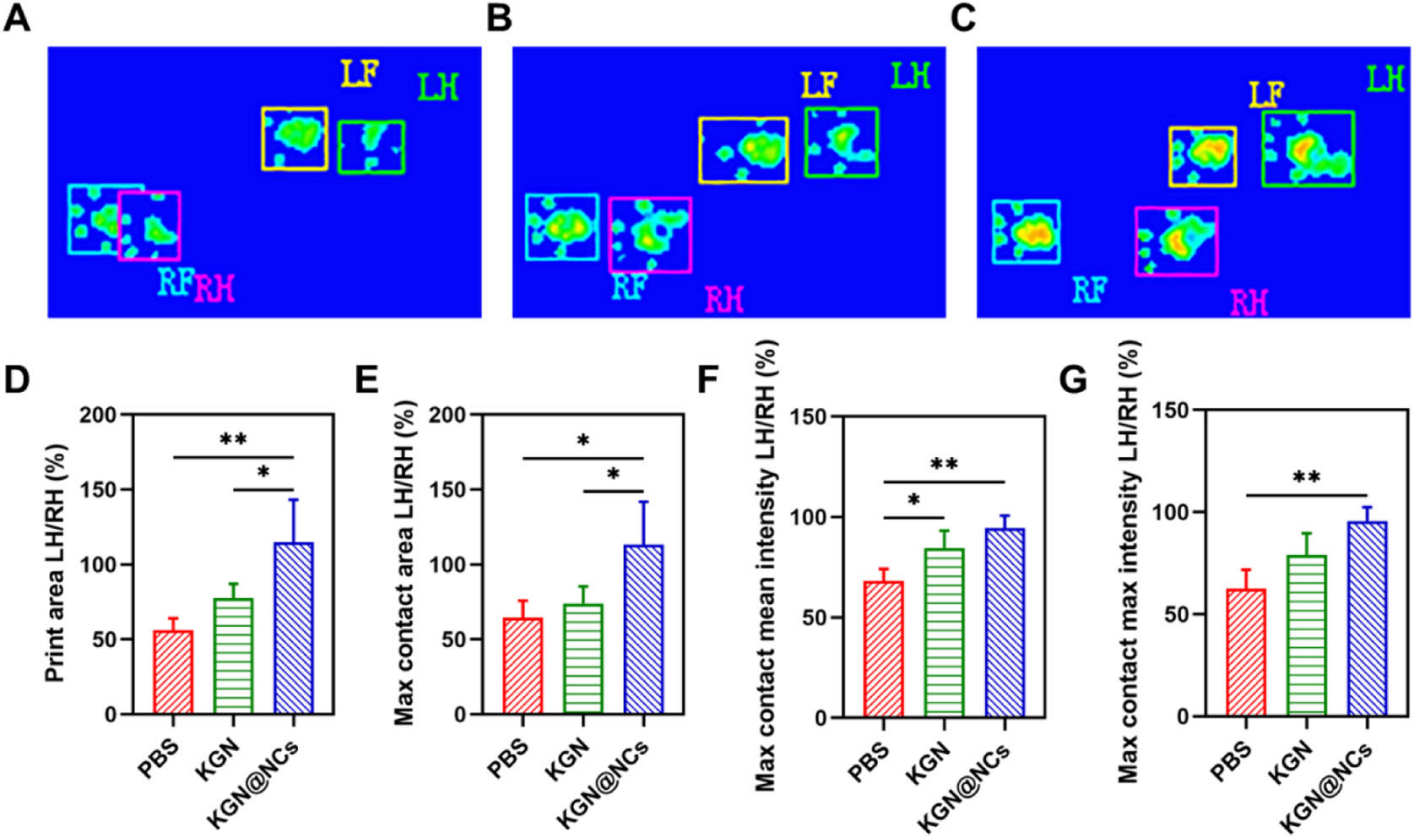
The gait parameters in a cartilage transplantation rat model. Representative gaits of the rats treated with PBS (**A**), KGN solution (**B**), and KGN@NCs (**C**). LH represented left hind paw and RH represented right hind paw. **D** The LH/RH ratio of print area (*n* = 6). **E** The LH/RH ratio of max contact area (*n* = 6). **F** The LH/RH ratio of max contact mean intensity (*n* = 6). **G** The LH/RH ratio of max contact max intensity (*n* = 6). All data were presented as mean ± SD. *p < 0.05, **p < 0.01

The in vivo therapeutic effects of KGN@NCs for cartilage repair was studied with a rat model of cartilage transplantation. The rats were randomly assigned into three groups and intra-articularly injected as follows (*n* = 6): PBS group, KGN group, and KGN@NCs group. After 4 weeks, Catwalk gait analyzing system was used for the evaluation of functional parameters of the gait of the rats (Fig. S3). From a cursory perspective, the print area and intensity of left hind paw obviously decreased in PBS group (Fig. 4A) than KGN group (Fig. 4B) and KGN@NCs group (Fig. 4C), suggesting the gait impairment was relieved when treated with KGN. Furthermore, functional parameters provided a quantitative analysis for these gaits. The print area, which is the surface area of the complete print, showed a significant decrease in PBS group (*p* < 0.01) and KGN group (*p* < 0.05) compared with KGN@NCs group (Fig. 4D). Meanwhile, the print area at max contact (max contact area) of KGN@NCs group was also higher than the other two groups (*p* < 0.05) (Fig. 4E). Moreover, the mean and max intensity at max contact (max contact mean/max intensity) exhibited a decrease in KGN group than KGN@NCs group, despite that the difference was not significant (*p* > 0.05) (Fig. 4F, G). Previous studies have demonstrated that the decrease of print area and intensity is a pain-related behavior in osteoarthritis and neurogenic disorders and represents attenuated motor functions [35–40]. Thus, KGN@NCs relieved the pain of the rats and helped with the recovery of the impaired motor functions.

### Histological assessment for cartilage repair

**Fig. 5:**
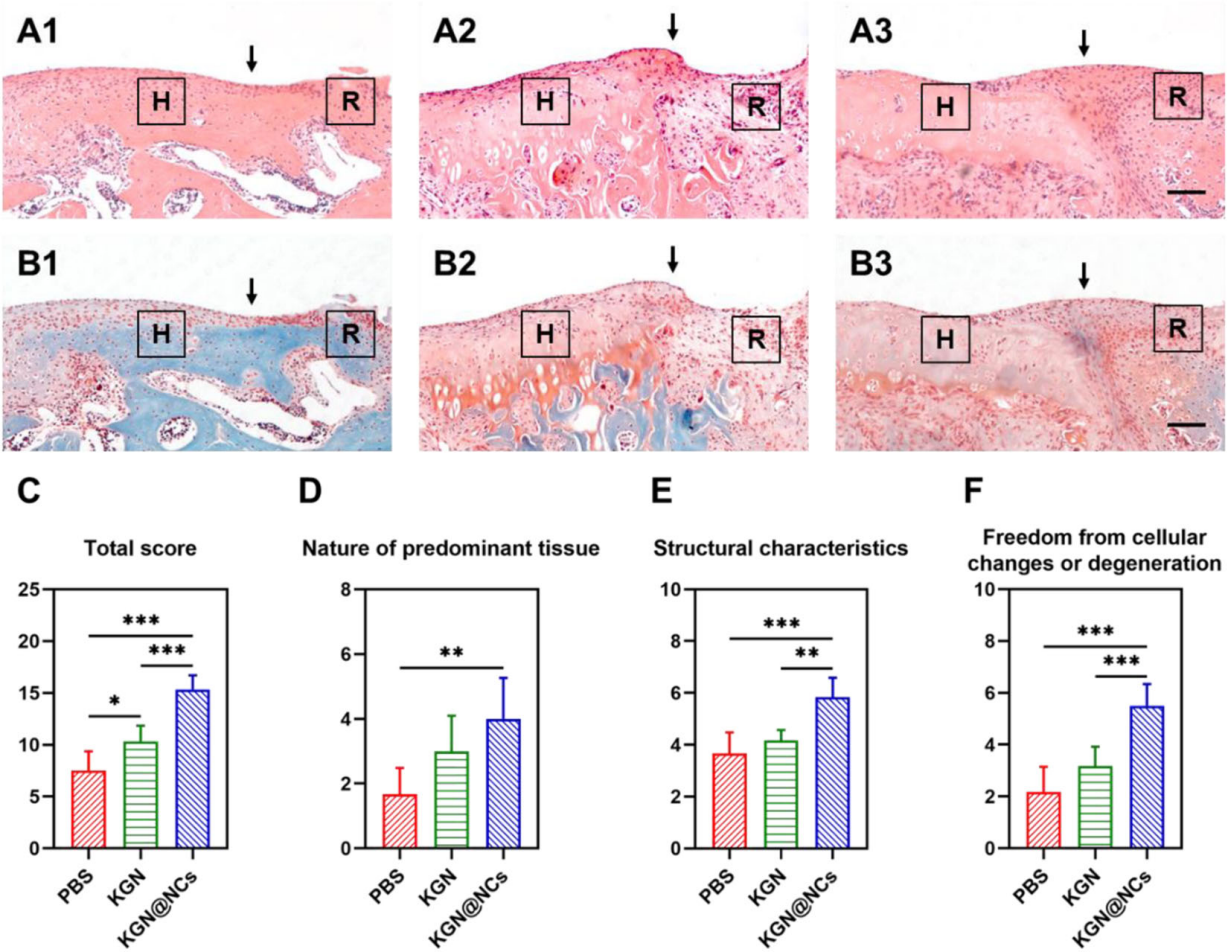
Histological staining and quantitative assessment of the repaired articular cartilage. Representative HE (**A**) and safranin O/fast green (**B**) staining for the cartilage of PBS group (A1, B1), KGN group (A2, B2), and KGN@NCs group (A3, B3) (scale bars, 100 μm). H represented the host cartilage and R represented the repaired cartilage. The black arrow indicated the border region of H and R. **C** The total score of O’Driscoll scoring system (*n* = 6). **D** The score of nature of predominant tissue (*n* = 6). **E** The score of structural characteristics (*n* = 6). **F** The score of freedom from cellular changes or degeneration (*n* = 6). All data were presented as mean ± SD. *p < 0.05, **p < 0.01, ***p < 0.001

To further investigate the detailed quality of the repaired tissues, the articular cartilage was observed for macroscopy after the rats were sacrificed. There was a circular gap with obvious degeneration in PBS group, while KGN group and KGN@NCs group were partly filled with the repaired tissues (Fig. S4). Then, the whole cartilage was sectioned and stained with HE and safranin O/fast green, and the border region between the host cartilage and the transplanted cartilage was evaluated. HE stained sections showed the gap regions of PBS group and KGN group were partly filled but without flat surfaces (Fig. 5A1, A2). In contrast, KGN@NCs group had a flatter surface with an obvious cluster of chondrocytes (Fig. 5A3), suggesting that the chondrogenic effect of KGN presented earlier than the other two groups. Moreover, sections stained with safranin O/fast green exhibited a layer of green staining underneath the articular surface in PBS group, which implied hyperplastic tissue calcification in the area of the cartilage (Fig. 5B1). In KGN group, weak safranin O-stained repaired cartilage was observed (Fig. 5B2), whereas there was obvious safranin O staining underneath the surface in KGN@NCs group, which revealed a higher level of aggrecan in the repaired cartilage (Fig. 5B3). Therefore, compared with single KGN, KGN@NCs were capable of generating the repaired cartilage with a flatter surface and a higher level of aggrecan.

Moreover, O’Driscoll scoring system for cartilage repair that contains three variable scores was introduced to accurately evaluate the repaired cartilage [33]. The total score of KGN@NCs group was higher than PBS group (*p* < 0.001) and KGN group (*p* < 0.001) (Fig. 5C). Firstly, nature of predominant tissue, which assessed the cellular morphology and matrix staining of safranin O, showed an increased score of KGN@NCs group compared with PBS group (*p* < 0.01) (Fig. 5D). Secondly, the score of structural characteristics for the assessment of surface regularity, structural integrity, thickness, and bonding was higher in KGN@NCs group than PBS group (*p* < 0.001) and KGN group (*p* < 0.01) (Fig. 5E). Thirdly, likewise, the score of freedom from cellular changes or degeneration increased in KGN@NCs compared with the other two groups (*p* < 0.001) (Fig. 5F). These quantitative results further indicated KGN@NCs promoted a higher quality of repaired cartilage than KGN.

For years, it has been a challenge for the newly repaired cartilage to heal with a regular structure in clinical cartilage defect therapy. The limited regularity of the repaired cartilage with the host cartilage induces a discontinuous surface and an imbalanced force that will lead to cartilage degeneration and motor disfunctions. In this study, compared with the single KGN, the prepared KGN@NCs generated a flatter surface and a higher level of aggrecan in the repaired cartilage at 4 weeks after the surgery in a rat model. Furthermore, the improvement of histology further improved the pain-related functional parameters of the rats to restore to a normal level in KGN@NCs group, which achieved the purpose of fast cartilage repair after the surgical treatment.

## Conclusions

In conclusion, the prepared KGN@NCs successfully loaded and released KGN to promote the viability of chondrocytes in vitro. Furthermore, KGN@NCs generated the repaired cartilage with a flatter surface and a higher level of aggrecan. Then, the quantitative analysis also showed a higher quality of the repaired cartilage in KGN@NCs group than KGN group. Moreover, with the intra-articularly delivered KGN@NCs, the motor functions of the rats recovered to a normal level compared with the single KGN. Overall, this drug delivery strategy consisting of NCs conjugated with therapeutic drug provided a promising perspective for cartilage repair.

## Supplementary information

Supplementary Information
