# Supplementary material for: Magnetic nanocarriers as a therapeutic drug delivery strategy for promoting pain-related motor functions in a rat model of cartilage transplantation
Source: J Mater Sci Mater Med. 2021 Mar 31;32(4):37. doi: 10.1007/s10856-021-06508-8 (PMC8012338; doi:10.1007/s10856-021-06508-8)
Supplement: Supplementary file 1 — Supplementary Information [file 10856_2021_6508_MOESM1_ESM.docx]

Supplementary information

Magnetic nanocarriers as a therapeutic drug delivery strategy for promoting pain-related motor functions in a rat model of cartilage transplantation

Xingyu Zhang1, Jianjun Yang2, Baochang Cheng3, Shenli Zhao4, Yao Li5, Hui Kang2, and Shiyi Chen1

These authors contributed equally: Xingyu Zhang, Jianjun Yang

Corresponding authors: Jianjun Yang (E-mail: yangjianjun0311@163.com), Shiyi Chen (E-mail: cshiyi@163.com)

1. Department of Sports Medicine, Huashan Hospital, Fudan University, Shanghai 200040, China

2. Department of Orthopaedics, Tenth People’s Hospital of Tongji University, Shanghai 200072, China

3. State Key Laboratory of Molecular Engineering of Polymers & Department of Macromolecular Science, Fudan University, Shanghai 200433, China

4. Department of Joint Surgery, Yangpu Hospital Affiliated to Tongji University, Shanghai 200082, China

5. Department of Orthopaedics, Tenth People’s Hospital of Nanjing Medical University, Shanghai 200072, China


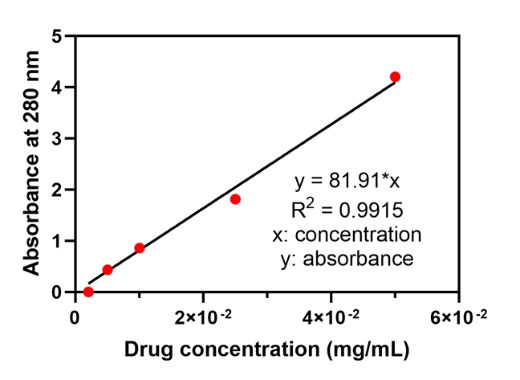


Fig. S1. The linear regression of KGN concentration and UV-vis absorbance.


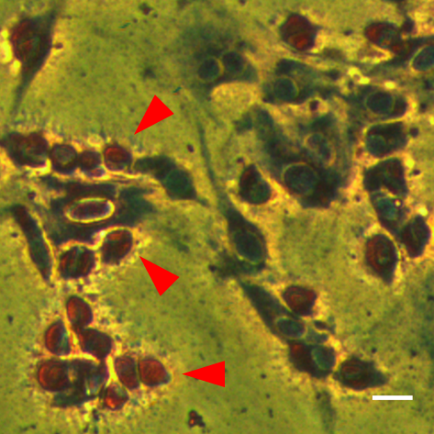


Fig. S2. The characterization of the isolated chondrocytes from the articular cartilage by toluidine blue staining (scale bar, 20 μm). Red arrowheads represented the stained chondrocytes.


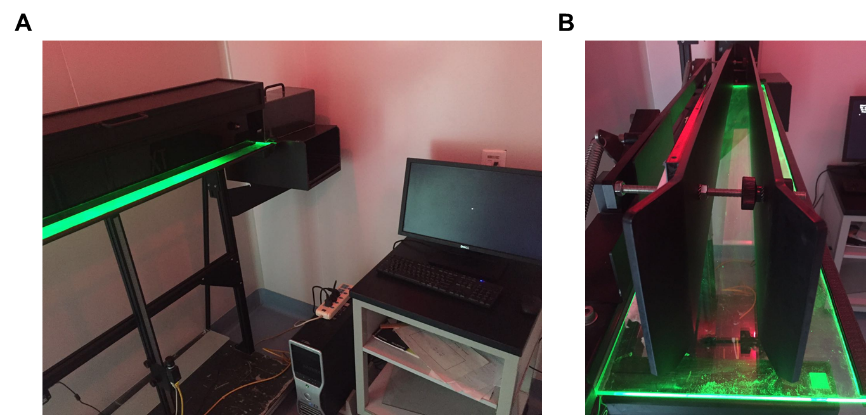


Fig. S3. Catwalk gait analyzing system. (A) The computer system. (B) The acquisition system.


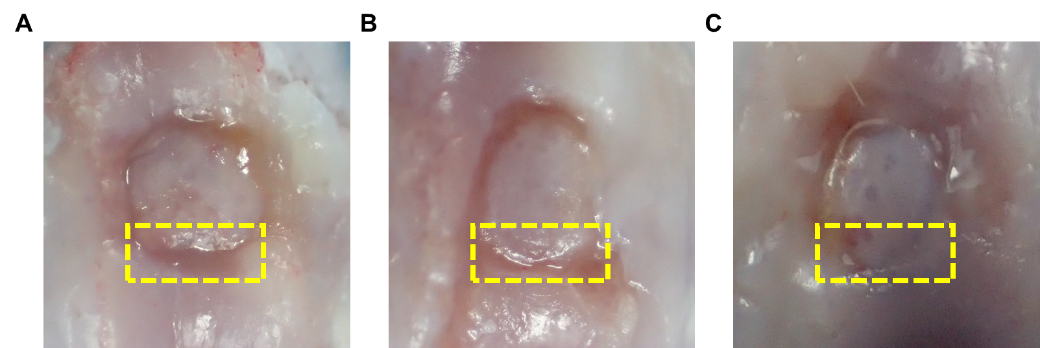


Fig. S4. The macroscopic evaluation of the repaired articular cartilage. A) PBS group. B) KGN group. C) KGN@NCs group. Yellow frames represented the observation area between the host cartilage and the transplanted cartilage.
